# Thermal (In)stability of Atropine and Scopolamine in the GC-MS Inlet

**DOI:** 10.3390/toxics9070156

**Published:** 2021-06-30

**Authors:** Gordana Koželj, Helena Prosen

**Affiliations:** 1 Institute of Forensic Medicine, Faculty of Medicine, University of Ljubljana, Korytkova 2, SI-1000 Ljubljana, Slovenia; 2 Faculty of Chemistry and Chemical Technology, University of Ljubljana, Večna pot 113, SI-1000 Ljubljana, Slovenia; helena.prosen@fkkt.uni-lj.si

**Keywords:** atropine, scopolamine, thermal stability, GC-MS inlet, degradation

## Abstract

The intoxication due to unintentional or intentional ingestion of plant material containing tropane alkaloids is quite frequent. GC-MS method is still widely used for the identification of these toxicologically important substances in human specimen. During general unknown analysis, high temperature of inlet, at least 270 °C, is commonly used for less volatile substances. Unfortunately, both tropanes are thermally unstable and could be overlooked due to their degradation. The temperature-related degradation of tropanes atropine and scopolamine was systematically studied in the inlet of a GC-MS instrument in the range 110–250 °C by increments of 20 °C, additionally also at 275 °C, and in different solvents. At inlet temperatures not higher than 250 °C, the degradation products were formed by elimination of water and cleavage of atropine’s ester bond. At higher temperatures, elimination of formaldehyde became predominant. These phenomena were less pronounced when ethyl acetate was used instead of methanol, while *n*-hexane proved unsuitable for several reasons. At an inlet temperature of 275 °C, tropanes were barely detectable. During systematic toxicological analysis, any tropanes’ degradation products should indicate the possible presence of atropine and/or scopolamine in the sample. It is not necessary to prepare thermally stable derivatives for confirmation. Instead, the inlet temperature can be decreased to 250 °C, which diminishes their degradation to a level where their detection and identification are possible. This was demonstrated in several case studies.

## 1. Introduction

Gas chromatography coupled to mass spectrometry (GC-MS) is still a method widely used in toxicology for the identification of unknown substances in human specimens. In comparison to liquid chromatography coupled to mass spectrometry (LC-MS), which is nowadays the prevalent technique also in toxicological laboratories, GC-MS has the main advantage of universal ionization method (electron ionization, EI), which is negligibly influenced by sample matrix and yields reproducible spectra that can be readily used to identify the compound either by comparison with mass spectral libraries or by direct interpretation. However, the applied oven temperatures are up to 350 °C, which means that the analysis is limited to small and volatile molecules (MW < 600 Da), which is the main disadvantage of GC-MS technique and the reason it is currently less often used than LC-MS. However, GC-MS still plays a major role in screening for the responsible substance in cases of intoxication of unknown origin, which cannot be readily accomplished by more compound-specific LC-MS methods. It should also be emphasized that GC-MS equipment is much more affordable compared even to a simple LC-MS system.

**Figure 1:**
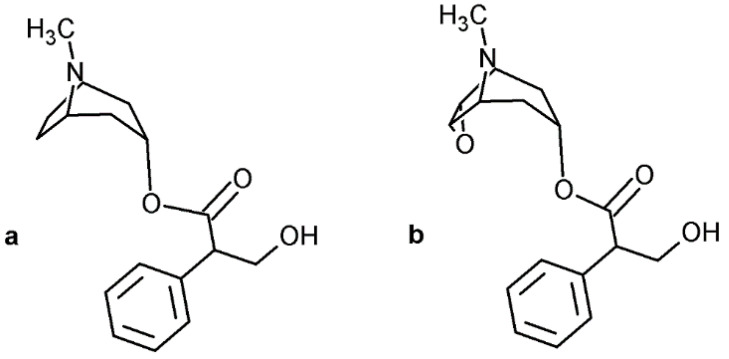
Chemical structure of atropine (**a**) and scopolamine (**b**).

Among toxicologically important substance, there are also two toxic alkaloids with a tropane skeleton, (–)-hyoscine (scopolamine, Figure 1) and (–)-hyoscyamine, which are found in plants of the family Solanaceae, e.g. *Atropa beladonna*, *Datura stramonium,* and *Hyoscyamus niger* [1]. The intoxication due to unintentional or intentional ingestion of plant material is quite frequent. (–)-Hyoscyamine is converted to a racemic mixture (±)-hyoscyamine (atropine, Figure 1) in the body after ingestion or during extraction from a biological sample. Frequently used methods for determination of atropine and scopolamine are gas chromatographic methods with mass-spectrometric detection [2,3,4,5]. As both alkaloids are thermally unstable, stable derivatives, such as trimethylsilyl [6,7,8], acetyl [5], or pentafluoropropyl [9], need to be prepared for a successful GC-MS determination. In order to avoid degradation problems, nowadays, liquid chromatography-mass spectrometry methods are used instead and are the methods of choice if quantitative analysis is required [10,11].

The concentrations of both tropanes in biological samples are quite low. For atropine, therapeutic concentrations are 0.002–0.025 mg/L, toxic from 0.03 to 0.1 mg/L, and lethal around or over 0.2 mg/L. Concentrations of scopolamine in blood are almost ten times lower, in the range of 0.0001 to 0.01 mg/L [12]. Half-time of atropine in blood is 2–4 h, the majority of the dose is excreted in urine in one day, of which 13–50% as original substance and the rest as metabolites. The toxic dose can be as little as 1 mg, while the lethal dose is 50–100 mg. Scopolamine has a longer half time of 2–6 h and is excreted mostly (95%) as metabolites in urine in approximately two days. Fatal poisonings are extremely rare [12].

Due to their low concentrations in biological fluids, the risk of overlooking the presence of tropane alkaloids increases with their degradation. They could be easily missed in the general unknown GC-MS screening in cases of suspected intoxication. Thus, our intention was to study the phenomenon of their thermal degradation at different temperatures and in different solvents to establish how both compounds can be successfully recognized from their thermal degradation products. Solutions in pure solvents were used to avoid potential matrix effects besides the studied effects. The successful identification by applying the findings of our study is demonstrated in several case studies.

## 2. Materials and Methods

### 2.1. Chemicals and Reagents

Methanol Chromasolv® was purchased from Sigma-Aldrich (Steinheim, Germany), *n*-hexane and ethyl acetate from Merck (Darmstadt, Germany).

Scopolamine hydrochloride (>99%) and atropine (USP testing specifications) were purchased from Sigma-Aldrich (St. Louis, MO, USA).

### 2.2. GC-MS Analysis

A Hewlett Packard 6890 Plus gas chromatograph with an Agilent 7683 series injector and a 5973 mass selective detector (Hewlett Packard, Palo Alto, CA, USA) were used for qualitative analyses. The compounds were separated on an HP-5MS capillary column (5%-phenyl-methylpolysiloxane, 30 m × 0.25 mm i.d., 0.25 μm film thickness). Helium was used as a carrier gas at a constant flow of 1 mL/min. The GC inlet temperature was changed from 110 °C to 250 °C by increments of 20 °C and finally to 275 °C, which is the temperature used in our routine general unknown screening. The inlet liner was Agilent splitless single taper, deactivated liner with glass wool (Agilent, Palo Alto, CA, USA, P/N: 5062-3587). The initial oven temperature of 60 °C was held for 2 min and then ramped at 20 °C/min to 300 °C with a final hold time of 15 min. The temperature of the transfer line was 280 °C. The ion source and quadrupole temperatures were 230 °C and 150 °C, respectively. The mass selective detector was used in EI scan mode, the ionization energy was 70 eV, and the electron multiplier voltage was set 200 V above autotune value. Data were collected for a splitless injection of 1.0 µL of each tropane in methanol, ethyl acetate and *n*-hexane in the range from 50 to 550 *m*/*z* at a rate of 2.9 scans/s. The software used was MSD Chemstation D.01.02.16.

### 2.3. Sample Preparation

The solutions of atropine and scopolamine base were prepared in methanol, ethyl acetate and *n*-hexane with the final concentration of 40 mg/L for methanol and 200 mg/L for the other two solvents.

## 3. Results and Discussion

### 3.1. Solutions of Atropine in Scopolamine in Different Solvents

**Figure 2:**
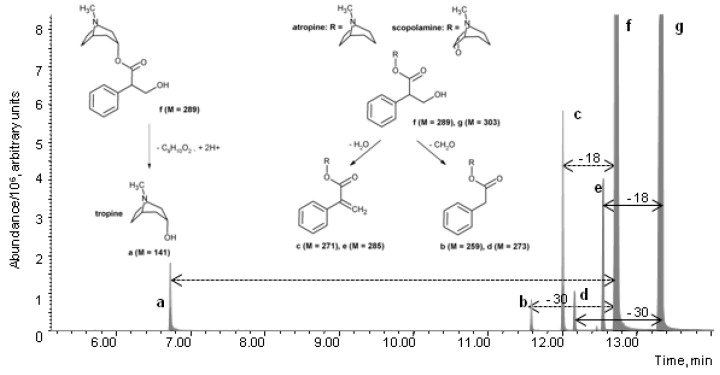
GC-MS chromatogram of atropine, scopolamine, and their thermolysis products with a tropane skeleton, obtained by extraction and merging of two ion currents (*m*/*z* 124–atropine related and *m*/*z* 94–scopolamine related) from TIC. The elimination of water (Δ*M*: −18) and formaldehyde (Δ*M*: −30) was observed in both tropanes while a significant cleavage of ester bond was seen only in atropine. The inlet temperature was 250 °C. Tropine (**a**), 3-phenylacetoxytropane (atropine-CH_2_O) (**b**), apoatropine (atropine-H_2_O) (**c**), 3-phenylacetoxyscopine (scopolamine-CH_2_O) (**d**), aposcopolamine (scopolamine-H_2_O) (**e**), atropine (**f**), and scopolamine (**g**).

In our practice, in the case of general unknown analysis, LLE or SPE extracts of biological samples are dissolved in methanol and afterwards, screened by the GC-MS method described in the Experimental Section 2.2. Methanol, being both a proton donor and acceptor, is used as a solvent for a variety of substances that are efficiently dissolved despite their frequently quite different polarity. The sample is vaporized in the inlet and transferred to a column at a low initial temperature where condensation of solvent and sample occurs. As the temperature is increased, the methanol evaporates and the sample is preconcentrated in the residual solvent within a narrow area. Analytes are vaporized from this area at the beginning of the column at higher oven temperatures. However, a high temperature of the injector is used to vaporize less volatile substances. Because of this, the occurrence of thermal decomposition products, methylated substances, and formaldehyde adducts is possible. Several mass spectra of such compounds are included in commercial mass spectra libraries (e.g., Pfleger/Maurer/Weber, NIST) but are not necessarily marked as such, and the recognition of their origin depends on the analyst’s knowledge and experience. Thermolysis is noticeable in atropine and scopolamine, as illustrated in Figure 2.

**Figure 3:**
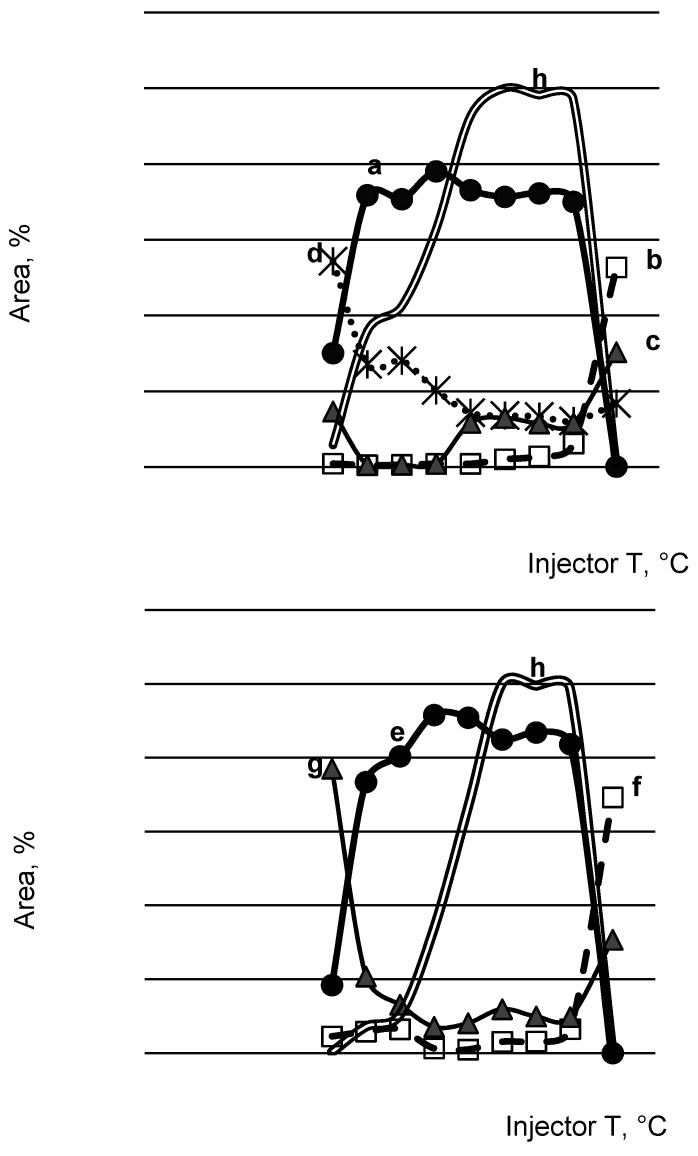
Influence of injector temperature on peak areas of atropine, scopolamine, and their main products of thermolysis, solvent methanol. Atropine (**a**), 3-phenylacetoxytropane (atropine-CH_2_O) (**b**), apoatropine (atropine-H_2_O) (**c**), tropine (**d**), scopolamine (**e**), 3-phenylacetoxyscopine (scopolamine-CH_2_O) (**f**), aposcopolamine (scopolamine-H_2_O) (**g**), sum of areas normalized to maximum value (**h**).

**Figure 4:**
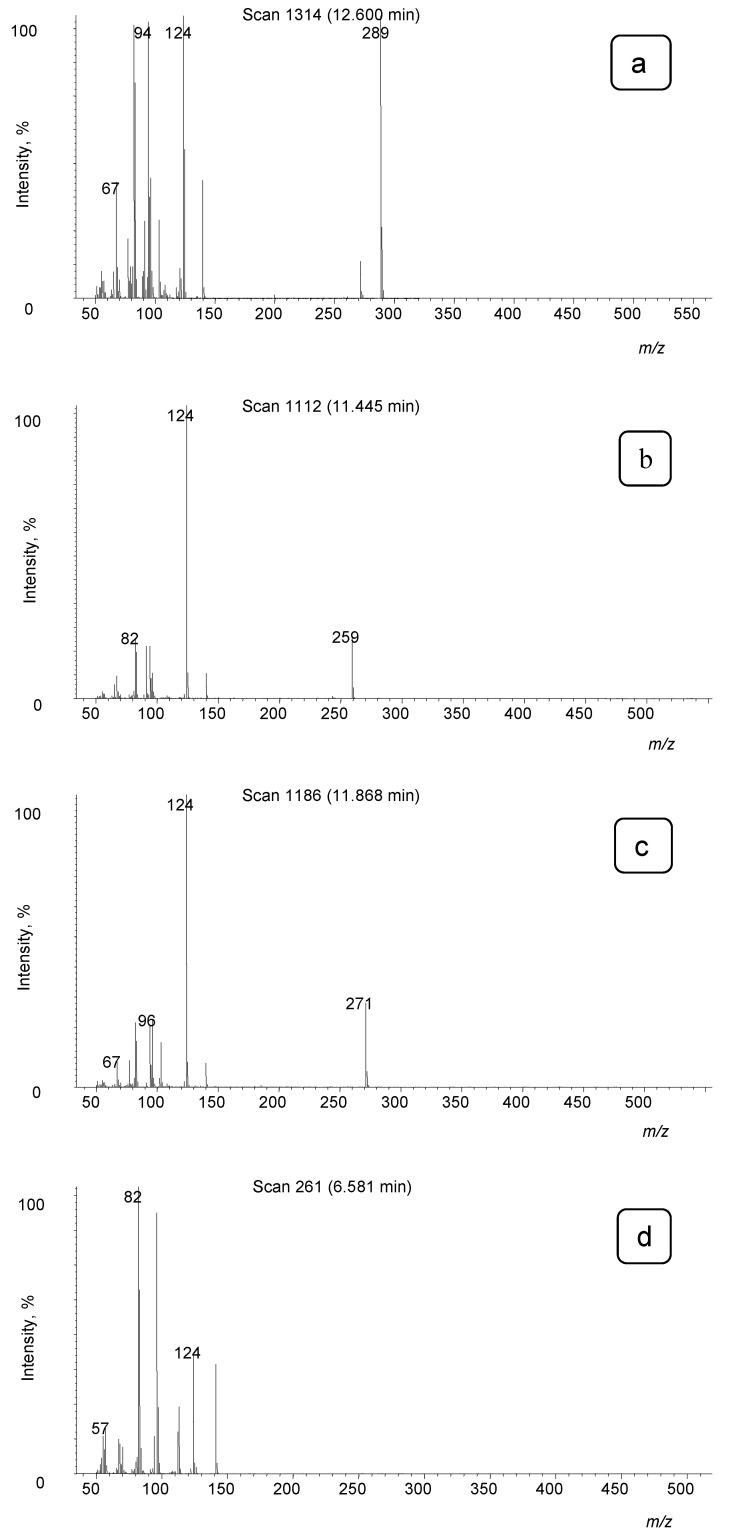

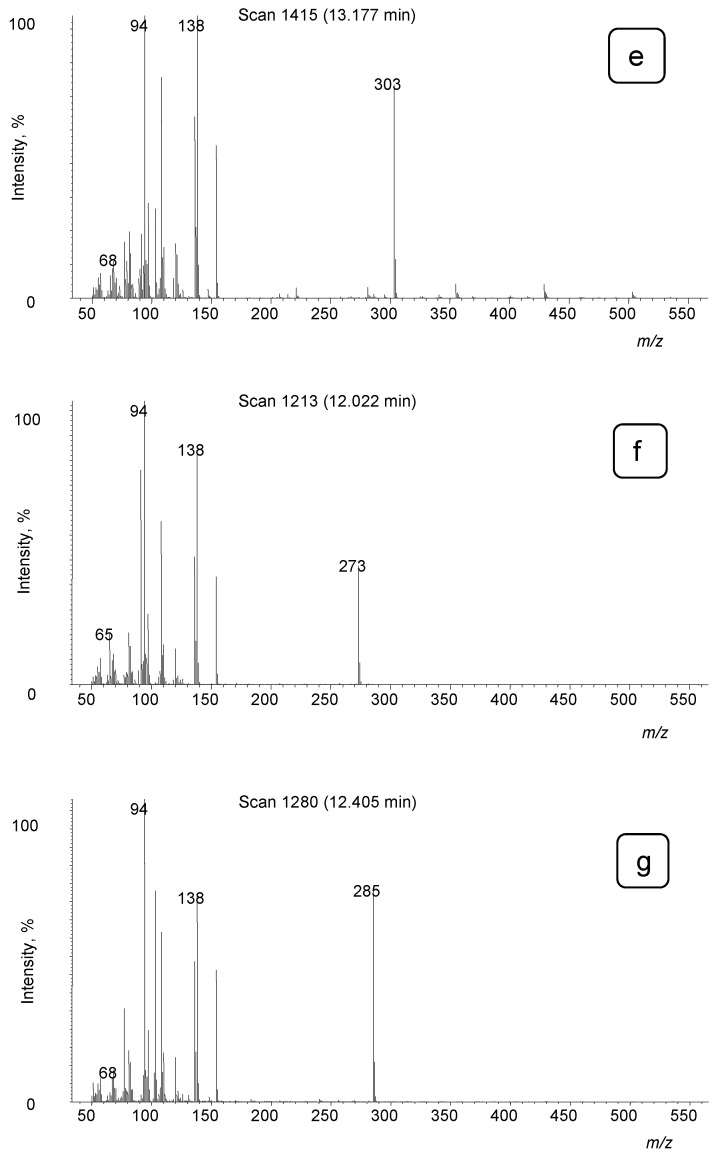
Mass spectra of atropine, scopolamine and their main products of thermolysis. Atropine (**a**), 3-phenylacetoxytropane (atropine-CH_2_O) (**b**), apoatropine (atropine-H_2_O) (**c**), tropine (**d**), scopolamine (**e**), 3-phenylacetoxyscopine (scopolamine-CH_2_O) (**f**), aposcopolamine (scopolamine-H_2_O) (**g**).

We were interested in the extent of thermal degradation at different conditions. Thus, the behavior of both substances in the solution with three solvents of different polarities was studied, with methanol being the most polar, less polar ethyl acetate, and *n*-hexane as the nonpolar one. Tropanes were also exposed to different injector temperatures. As an example, only results for methanol as solvent are presented in Figure 3, while additional results for other solvents are given in Supplementary Materials, Figure S1 and Table S1. In the temperature range from 100 to 250 °C, the thermal energy is sufficient only for low-energy reactions such as functional group elimination at the end of the chain [13]. In our case, this corresponds to elimination of water from the atropine and scopolamine molecules in the GC inlet and the cleavage of the ester bond, the latter observed only in atropine. Atropine is more degradable than scopolamine. The main structural difference lies in the cyclic ether bond, which could be the reason for increased degradability. In the temperature range from 250 to 500 °C, there is enough thermal energy to break the chemical bonds with the highest energy [13]. Consequently, also the elimination of formaldehyde becomes important for tropanes at higher inlet temperatures. Successive elution of tropanes and their degradation products from the chromatographic column, corresponding to their specific physico-chemical properties, is proof of thermal degradation taking place in the inlet of a gas chromatograph. The corresponding mass spectra are presented in Figure 4.

The degradation phenomena were more pronounced in methanol. With temperatures below 250 °C, the elimination of water was predominant compared to formaldehyde elimination. Both processes were comparable but proceeded to a negligible extent in ethyl acetate again up to 250 °C. At 275 °C, atropine and scopolamine were almost completely degraded both in methanol and ethyl acetate. In the case of *n*-hexane as the solvent, the situation was fully unpredictable, rendering different and randomly changing ratios of peak areas, with comparable areas for atropine, scopolamine, and their degradation products and giving poor response for parent compounds. Described observations could have arisen from the properties of the applied solvents, which contributed to the degradation process. The most pronounced influence was from methanol, being both a proton acceptor and donor, and less from ethyl acetate, which is only a proton acceptor. *n*-Hexane is neither a proton acceptor nor proton donor. Moreover, both analytes are poorly soluble in it. Low solubility could cause their wider dispersion upon entering the GC column (at 60 °C) and lower preconcentration at the beginning of the column, leading to higher exposure to heat and consequently, a higher relative amount of thermal decomposition products, which was actually observed. To summarize: Methanol as solvent caused the highest degree of degradation both at temperatures below or above 250 °C. Ethyl acetate promoted degradation only at temperatures above 250 °C, while for *n*-hexane, no behavioral pattern could be established.

In the next step, we wanted to investigate the influence of analyte concentration on the extent of thermal degradation. We chose the following experimental conditions: The solvent was ethyl acetate and not methanol with a more pronounced influence on degradation, the inlet temperature was lowered to 250 °C to preserve the majority of parent compounds. Results for the solutions with concentration 200 and 2 mg/L (hundred-times dilution) were compared with those obtained for the same solutions at inlet temperature 275 °C (Table 1 and Table 2). Atropine was significantly more degraded at the higher inlet temperature than scopolamine and additionally with dilution.

### 3.2. Application on Real Samples

The GC-MS method described in Experimental Section 2.2 has been used in our laboratory for more than 25 years as a general screening method in forensic, clinical, DUI, and drugs of abuse cases. From the very beginning, we have used a lower inlet temperature of 275 °C compared to usually higher reported *T* of screening procedures, for instance, 280 °C [14]. The decision was based on observed degradations of some compounds (for example, oxazepam, ß-blockers) as well as on cases with problematic or even unsuccessful primary identification. Despite the analysis of several thousands of cases per year, atropine was present in a very small number of poisonings and scopolamine even in less. Some identification problems of atropine and scopolamine will be presented in the following case studies.

- Case study 1

A young male was found irresponsive, hallucinating. After successful treatment in the hospital, he admitted taking tea prepared from thorn-apple (*Datura stramonium*), containing atropine and scopolamine. GC-MS toxicological analysis of extracted urine confirmed the presence of both alkaloids and degradation products of scopolamine, namely scopolamine-H_2_O and scopolamine-CH_2_O. All compounds were reliably identified with a library search algorithm. Predominant chromatographic peaks were, in this case, degradation products of scopolamine, outstanding was scopolamine-H_2_O presenting 78% of a sum of areas, while for scopolamine, it was only 2%. The peak area of atropine was small compared to scopolamine. Concentrations of parent compounds were subsequently determined with a validated LC-MS/MS method [10]. The concentration of atropine in blood was 0.019 mg/L and in urine 0.30 mg/L, while concentrations of scopolamine were 0.011 and 0.95 mg/L, respectively.

- Case study 2

A young male was treated in the hospital emergency department after drinking tea. The same GC-MS method was used as above for identification of toxic compounds in extract of urine sample and extract of tea sample. Library search hits for tea extract were acetylated homatropine, atropine-H_2_O, and acetylated atropine, the latter at a significantly inappropriate retention time. Based on the presence of atropine degradation products and heteroanamnestic data, typical mass fragments were extracted (*m*/*z* 84, 124, 289, 94, 138, and 303), leading to the confirmation of atropine and scopolamine as well as their degradation products formed by elimination of water and formaldehyde. Library search of mass spectra of peaks of urine extract gave only a hit for atropine-CH_2_O, probably due to high background, and no other toxicologically relevant compounds were identified. With the extraction of typical mass fragments from the recorded mass spectra, the presence of atropine, atropine-H_2_O and atropine-CH_2_O was confirmed (estimated percentage of the sum of areas were 33%, 7%, and 60%, respectively), as well as the presence of scopolamine, scopolamine-H_2_O, and scopolamine-CH_2_O (estimated percentage of the sum of areas were 64%, 16%, and 20%, respectively).

- Case study 3

Buckwheat grain and flour (*Fagopyrum* sp., Polygonaceae) have an important role in the national Slovenian cuisine. Among various traditional dishes, a dish named “ajdovi žganci” is very popular (translated in English as “buckwheat spoonbread”). In September 2003, a mass food poisoning accident with the symptoms of a classic anticholinergic syndrome occurred in Slovenia. The National Institute of Public Health of Slovenia established an *ad hoc* self-reporting scheme and identified 73 cases with symptoms of tropane alkaloid toxicity. All intoxicated persons had consumed buckwheat flour food products within the last few hours. About 20 samples of disputable buckwheat products were analyzed by the presented GC-MS method after extraction of 30 g of material. All samples were dissolved in methanol and due to considerable amount of sample the presence of tropane alkaloids atropine and scopolamine was confirmed by library search. Degradation products of both compounds due to elimination of water and formaldehyde were identified after extraction of typical mass fragments from mass spectra, as well as traces of tropine. In the few delivered samples of blood and urine, tropanes or their degradation products were not identified. Further macroscopic examination of whole buckwheat grain revealed the presence of seeds of thorn-apple. At that time, temporary maximum residue levels (MRLs) of tropane alkaloids were established. To verify them, a study with volunteers was realized, and for that purpose, a validated LC-MS/MS method was developed [10,15].

- Case study 4

GC-MS analyses of blood serum samples taken from patients at the intensive care unit occasionally reveal the presence of atropine and/or atropine-H_2_O in extracts as a result of medical treatment.

Although the predominant processes during thermal exposure of tropanes depend on experimental parameters, good repeatability of measurements cannot be expected, making the method inappropriate for quantitative purposes. On the other hand, the thermal degradation products can offer additional valuable information while identifying compounds in the case of a general unknown analysis. In our practice, most often, the presence of dehydrated atropine or scopolamine triggers further appropriate analyses for the confirmation of their parent compounds. Depending on other circumstances, especially if other toxicologically important substances are present, one of the following possibilities is selected for further analysis: Inlet temperature is set to 250 °C, acetylated products are additionally prepared and identified, or the LC-MS/MS method is applied.

## 4. Conclusions

GC-MS methods have been used for decades for identification and quantitation of countless compounds occurring in our everyday life. For quantitative purposes, these methods are validated. One of the reasons for the occurrence of non-linearity of calibration curve for target parent compound can be temperature-induced degradation in the analytical instrument. In such cases, methods are appropriately corrected so that they fulfil the expected validation demands for parent compounds, but degradation products are usually not further studied.

The greatness of GC-MS methods still lies in their screening potential. Using the accepted standard experimental parameters for acquiring mass spectra enables the transfer of their electronic version between different instruments and laboratories. That enables the use of extensive, excellent mass spectra libraries in combination with RI values for identification even without having the pure standard of the compound. Combining these collections with nowadays powerful data analysis tools enables even small laboratories, for which buying a vast array of standards is financially difficult, to successfully perform identifications. Powerful tools, on the other hand, can lead to less focused, superficial interpretation, or diminished critical evaluation and observation of analytical results. For instance, even if the degradation of a particular compound is noticed, the impact of the process on the final result is seldom evaluated. In clinical toxicology, intoxicated patients are often treated according to their symptoms and signs, thus the plasma concentration of the particular compound is usually not sought at the very first moment or not at all. However, any information about the presence of toxic compounds, metabolites, or degradation products can be of importance. On the basis of such results, other confirmation or quantitation methods can be used. The same applies to questionable or deficient results in forensic cases, DUI, and drug abuse, where there is longer affordable time to conclude the analyses.

Some of the potentially toxic compounds which are identified in extracts of biological samples by the GC-MS method are thermally unstable. Tropanes atropine and scopolamine also belong to this group. Solutions of tropanes in pure solvents were used in this study to avoid potential additional matrix effects besides the temperature and solvent effects. We have observed that the extent of their degradation depends on the inlet temperature and also on the solvent used for the dissolution of the sample extract. The major degradation products formed in the inlet and observed up to 250 °C for both tropanes occurred after water elimination and atropine’s ester bond cleavage. At higher temperatures, elimination of formaldehyde became predominant and parent compounds atropine and scopolamine were barely detectable. With temperatures below 250 °C, less degradation was observed in the solvent ethyl acetate compared to methanol, while the behavior in *n*-hexane was shown to be unpredictable. Atropine is more thermally sensitive and thus more degraded than scopolamine. Thus, any estimation, that structurally very similar compounds are behaving in a similar manner, would be unjustified without actual experiments. The presence of tropanes and their degradation products should be considered during systematic toxicological analysis. If degradation products are observed, the temperature of the inlet can be lowered to 250 °C to allow for the detection and identification of parent compounds in a simpler manner compared to the time-consuming preparation of their derivatives. In the presented case studies, a comparable pattern of tropanes’ degradation was recognized and used for the identification of analytes.

## Figures and Tables

**Table 1:** Influence of inlet temperature and concentration on the ratio between atropine and its degradation products in ethyl acetate presented as percentage of a sum of areas.

Inlet *T* (°C)	Concentration (mg/L)	Atropine (%)	Atropine-H_2_O (%)	Atropine-CH_2_O (%)	Tropine (%)
275	200	48.1	49.6	0.5	1.8
	2	19.7	20.1	10.1	50.1
250	200	90.7	1.6	0.7	7.0
	2	91.2	2.0	2.6	4.2

**Table 2:** Influence of inlet temperature and concentration on the ratio between scopolamine and its degradation products in ethyl acetate presented as percentage of a sum of areas.

Inlet *T* (°C)	Concentration (mg/L)	Scopolamine (%)	Scopolamine-H_2_O (%)	Scopolamine-CH_2_O (%)
275	200	87.9	5.0	7.1
	2	91.2	6.0	2.8
250	200	95.9	2.6	1.5
	2	96.5	3.5	0.0

## Data Availability

The data presented in this study are available on request from corresponding author.

## Supplementary Materials

The following are available online at https://www.mdpi.com/article/10.3390/toxics9070156/s1, Figure S1: Influence of injector temperature on peak areas of atropine, scopolamine and their main products of thermolysis in three solvents: methanol, ethyl acetate and *n*-hexane, Table S1: Relation between sums of areas of particular tropane and main degradation products in different solvents at different injector *T*.

## References

[1] Dewick P.M. (2009). Medicinal Natural Products: A Biosynthetic Approach.

[2] Dräger B. (2002). Analysis of tropane and related alkaloids. J. Chromatogr..

[3] Aehle E., Drager B. (2010). Tropane alkaloid analysis by chromatographic and electrophoretic techniques: An update. J. Chromatogr. B.

[4] Beyer J., Drummer O.H., Maurer H.H. (2009). Analysis of toxic alkaloids in body samples. Forensic Sci. Int..

[5] El Bazaoui A., Bellimam M.A., Soulaymani A. (2011). Nine new tropane alkaloids from *Datura stramonium* L. identified by GC/MS. Fitoterapia.

[6] Namera A., Yashiki M., Hirose Y., Yamaji S., Tani T., Kojima T. (2002). Quantitative analysis of tropane alkaloids in biological materials by gas chromatography-mass spectrometry. Forensic Sci. Int..

[7] Oertel R., Richter K., Ebert U., Kirch W. (1996). Determination of scopolamine in human serum by gas chromatography-ion trap tandem mass spectrometry. J. Chromatogr. B.

[8] Papoutsis I., Nikolau P., Spiliopoulou C., Pistos C., Stefanidou M., Athanaselis S. (2012). A simple and sensitive GC/MS method for the determination of atropine during therapy of anticholinesterase poisoning in serum samples. Drug Test. Anal..

[9] Saady J., Poklis A. (1989). Determination of Atropine in Blood by Gas-Chromatography Mass-Spectrometry. J. Anal. Toxicol..

[10] Koželj G., Perharič L., Stanovnik L., Prosen H. (2014). Simple validated LC-MS/MS method for the determination of atropine and scopolamine in plasma for clinical and forensic toxicological purposes. J. Pharm. Biomed. Anal..

[11] Zhang P.T., Li Y.M., Liu G.H., Sun X.M., Zhou Y.T., Deng X.J., Liao Q.F., Xie Z.Y. (2014). Simultaneous determination of atropine, scopolamine, and anisodamine from *Hyoscyamus niger* L. in rat plasma by high-performance liquid chromatography with tandem mass spectrometry and its application to a pharmacokinetics study. J. Sep. Sci..

[12] Moffat A.C., Osselton M.D., Widdop B. (2004). Clarke’s Analysis of Drugs and Poisons.

[13] Moldoveanu S.C. (1998). Analytical Pyrolysis of Natural Organic Polymers.

[14] Maurer H.H., Pfleger K., Weber A.A. (2011). Mass Spectral Library of Drugs, Poisons, Pesticides, Pollutants: And Their Metabolites, Experimental Section.

[15] Perharič L., Koželj G., Družina B., Stanovnik L. (2012). Risk assessment of buckwheat flour contaminated by thorn-apple (*Datura stramonium* L.) alkaloids: A case study from Slovenia. Food Addit. Contam. A.

