# Supplementary material for: Thermal (In)stability of Atropine and Scopolamine in the GC-MS Inlet
Source: Toxics. 2021 Jun 30;9(7):156. doi: 10.3390/toxics9070156 (PMC8309868; doi:10.3390/toxics9070156)
Supplement: Supplementary file 1 [file toxics-09-00156-s001.zip › toxics-1266936-supplementary.pdf]

toxics

Supplementary Materials: Thermal (In)stability of Atropine and Scopolamine in the GC-MS Inlet

Gordana Koželj and Helena Prosen

Table S1. Relation between sums of areas of particular tropane and main degradation products in different solvents at different injector *T*.

Relation between sums of areas of atropine and main products in different solvents at different injector *T*.

Injector *T* (°C)	Areas in ethyl acetate/areas in methanol (%)	Areas in *n*-hexane/areas in methanol (%)
110	0.035	0.005
130	0.018	0.119
150	0.037	0.004
170	0.053	0.000
190	0.043	0.000
210	0.039	0.002
230	0.037	0.005
250	0.035	0.000

Relation between sum of areas of scopolamine and main products in different solvents at different injector *T*.

Injector *T* (°C)	Areas in ethyl acetate/areas in methanol (%)	Areas in *n*-hexane/areas in methanol (%)
110	0.017	0.078
130	0.012	0.342
150	0.020	0.008
170	0.017	0.000
190	0.014	0.001
210	0.010	0.001
230	0.009	0.001
250	0.009	0.000

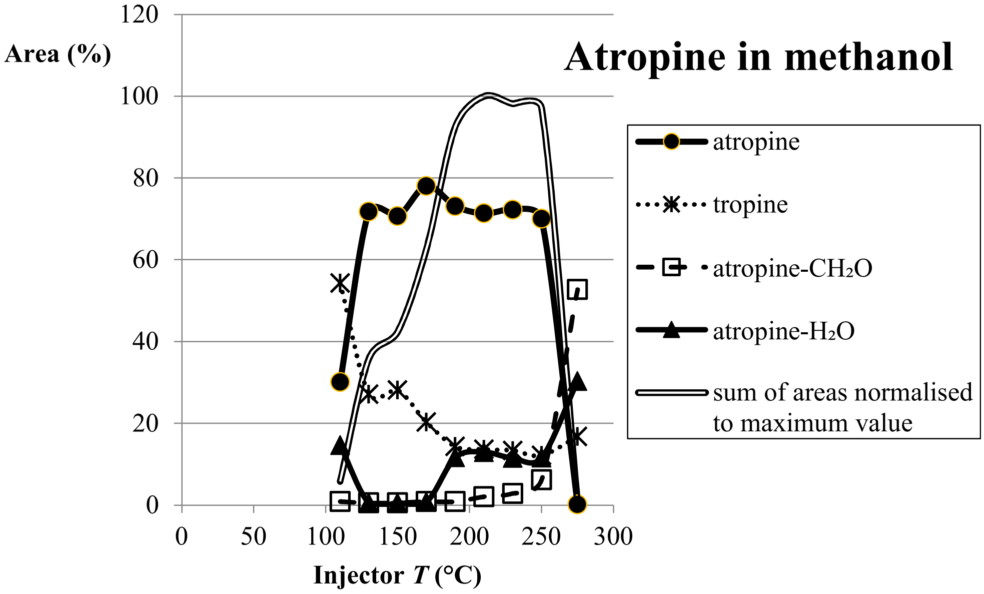
Atropine in methanol
Area (%)
120
100
80
60
40
20
0
0 50 100 150 200 250 300
Injector T (°C)
atropine
tropine
atropine-CH₂O
atropine-H₂O
sum of areas normalised to maximum value

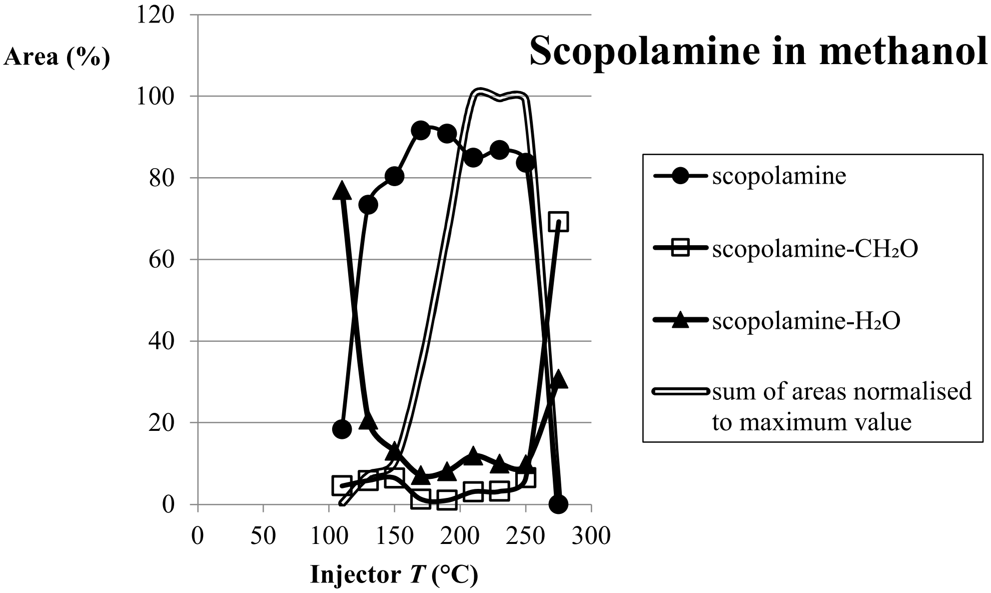
Scopolamine in methanol
Area (%)
120
100
80
60
40
20
0
0 50 100 150 200 250 300
Injector T (°C)
scopolamine
scopolamine-CH₂O
scopolamine-H₂O
sum of areas normalised to maximum value

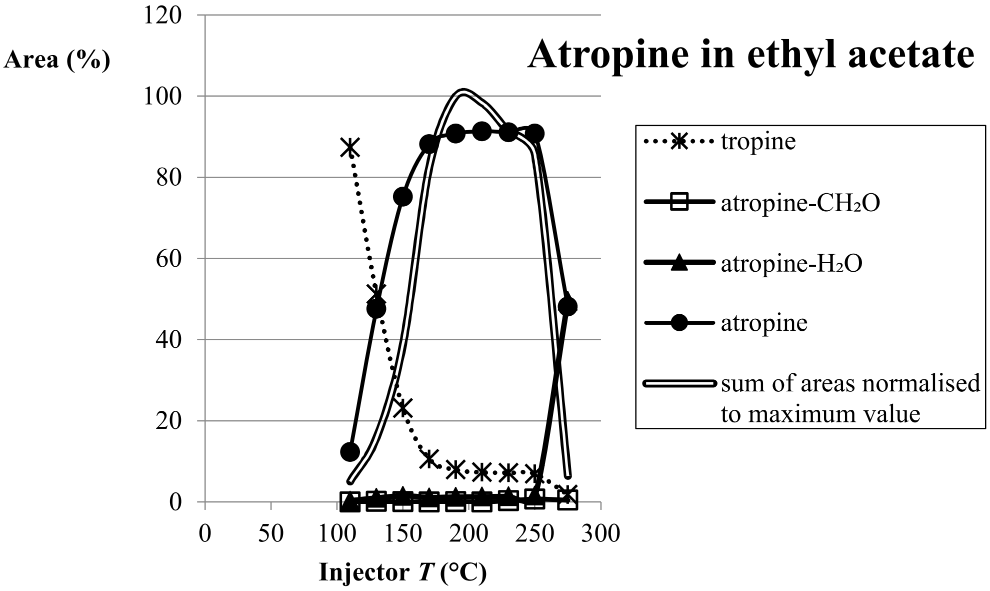
Atropine in ethyl acetate
Area (%)
120
100
80
60
40
20
0
0 50 100 150 200 250 300
Injector T (°C)
tropine
atropine-CH2O
atropine-H2O
atropine
sum of areas normalised to maximum value

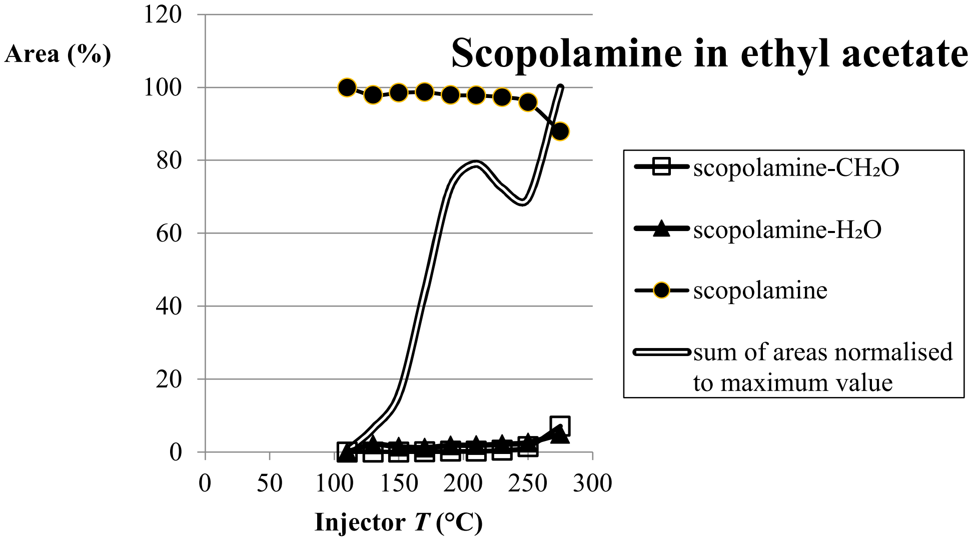
Scopolamine in ethyl acetate
Area (%)
120
100
80
60
40
20
0
0 50 100 150 200 250 300
Injector T (°C)
scopolamine-CH2O
scopolamine-H2O
scopolamine
sum of areas normalised to maximum value

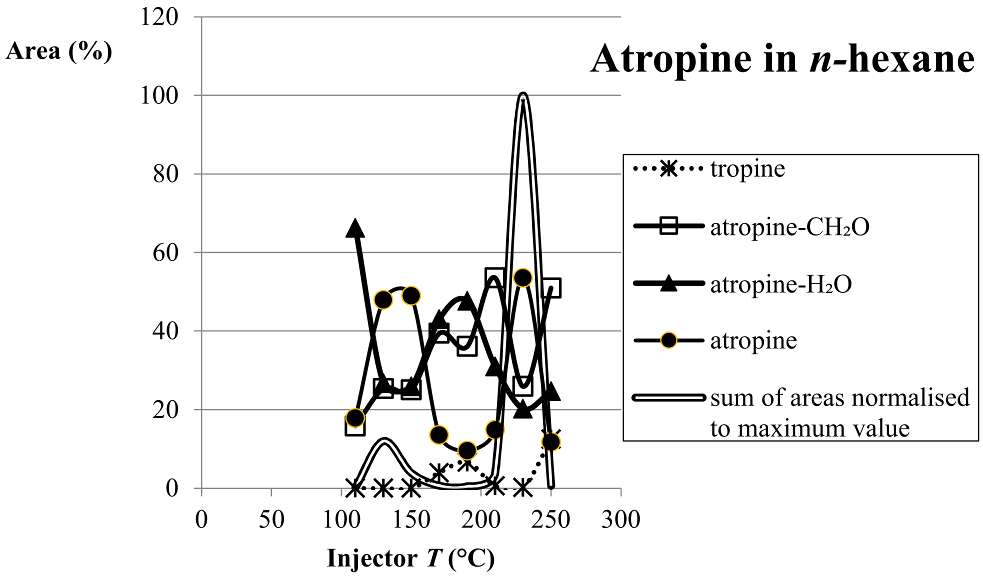

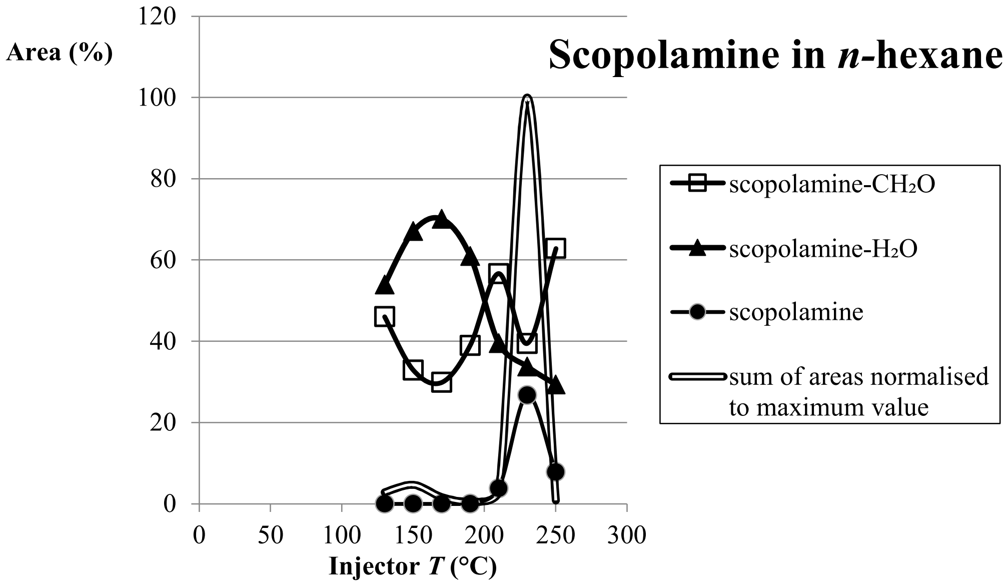

Figure S1. Influence of injector temperature on peak areas of atropine, scopolamine and their main products of thermolysis in three solvents: methanol, ethyl acetate and *n*-hexane.
